# Development of a scale to measure nurses’ information sharing practices during care transitions for older adults with high healthcare needs: a cross-sectional study

**DOI:** 10.3389/fpubh.2026.1938148

**Published:** 2026-09-10

**Authors:** Chihiro Kimura, K. A. T. M. Ehsanul Huq, Yuko Tsumoto, Michiko Moriyama

**Affiliations:** 1 Nursing Advancement Division, Unnan City Hospital, Unnan, Shimane, Japan; 2 Graduate School of Biomedical and Health Sciences, Hiroshima University, Hiroshima, Japan; 3 School of Nursing, Faculty of Medicine, Shimane University, Izumo, Japan

**Keywords:** care transition, hospital nurses, information sharing, older adults, scale development

## Abstract

**Background:**

Care transitions refer to the movement of patients between healthcare providers and/or care settings, such as from hospital to home. Continuous interventions, responses to unexpected changes during care transitions, and discharge support programs have been implemented to improve care transitions. However, a scale to measure information-sharing practices that address uncertainty in care transitions and differences in stakeholder perspectives has yet to be developed. Therefore, this study aimed to develop and evaluate the validity and reliability of an instrument to assess information-sharing practices among nurses during care transitions from hospital to home, with a focus on the discharge of older adults with high healthcare needs.

**Methods:**

The study involved several stages to develop the Care Transition Information Sharing Practice Scale (CT-ISPS). At first, the scale construct was defined based on the literature reviews and reviewed by experts, 36 preliminary items were generated. A cross-sectional survey was conducted to assess the scale’s validity and reliability. Nurses with ≥5 years of clinical experience in discharge support working at randomly selected hospitals were enrolled. The questionnaire included the newly developed CT-ISPS, the Interprofessional Collaboration Behavior Scale (ICBS) to assess convergent validity, and nurses’ demographic characteristics.

**Results:**

A total of 353 valid responses were obtained from the nationwide survey. Item analysis and exploratory factor analysis resulted in a 23-item scale comprising 3 factors: (1) knowledge sharing to support adaptation, (2) sharing patients’ holistic status to set direction, and (3) sharing external factors for anticipation. Confirmatory factor analysis demonstrated a mixed-model fit (CFI = 0.91, RMSEA = 0.093). Cronbach’s alpha coefficients for the factors ranged from 0.83 to 0.95, indicating adequate internal consistency. The correlation coefficient supported convergent validity between CT-ISPS and ICBS.

**Conclusion:**

The scale demonstrated preliminary evidence of structural validity, construct validity, and internal consistency in assessing information-sharing practices during care transition at discharge among hospital nurses. Future research should further examine its validity among home-care providers and other stakeholders involved in care transitions.

## 1 Introduction

Rapid population aging and ongoing healthcare reforms have led Japan to develop a community-based integrated care system that strengthens coordination across healthcare, long-term care, and social welfare services (1). These services have been promoted through a transformative social framework, including community care meetings, information-sharing sheets, and regional care pathways (1). Older adults with high healthcare needs require frequent medical and nursing care because of complex conditions, including critical and unstable symptoms and the use of medical devices (2). Regional collaboration, as envisioned in the community-based integrated care system, is particularly important for those older adults with chronic conditions, who frequently transition between hospitals, long-term care facilities, and home-based services (3). Ensuring smooth care transitions across these settings is therefore essential for improving continuity of care and health outcomes for older adults.

Care transitions refer to changes in care providers or care settings and are associated with elevated patient safety risks, including adverse drug events, complications, and hospital readmissions (4). To address these risks, multicomponent interventions have been implemented, including identifying high-risk patients, promoting communication between hospitals and community providers, and educating patients and caregivers, such as family members and friends (5). Evidence shows that these multicomponent interventions have improved outcomes, including reduced readmissions, improved quality of life, increased patient satisfaction, and lower healthcare costs (6). In Japan, policy initiatives have strengthened the discharge process and cross-setting continuity of care by promoting discharge support nurses and multidisciplinary discharge conferences for high-risk patients (7).

Information sharing in care transition refers to the practice of identifying and organizing the information needed (5), translating it into a form that is understandable and actionable (8), and communicating and sharing it bidirectionally among stakeholders including patients, caregivers, and care providers (8, 9). Information sharing is a key determinant of the safety and quality of care transitions (9, 10), with interventions including discharge medication summaries, self-care education, and information tailored to the individual needs (11). However, the Commonwealth Fund International Health Policy Survey across 11 high-income countries found that poor discharge communication was common among older adults and was most often related to medication information not being discussed (12). Other studies in Nordic capitals found that even within broadly similar welfare states, differences in organizational structure and in access to shared patient records shaped the completeness of information exchanged between hospitals and home-care services (13). These findings indicate that inadequate information sharing during care transitions is a persistent global challenge that can compromise patient safety and the continuity and quality of care.

Care transitions involve complex interactions among multiple stakeholders across different care settings. They require navigating physical, organizational, and sociocultural boundaries, which creates inherent uncertainty and unpredictability (14). These challenges are especially pronounced for older adults with high healthcare needs as symptoms, living environments, caregiving arrangements, and social resources interact dynamically, making outcomes difficult to predict (15). Such complexity highlights the need for healthcare systems to adapt to changing circumstances. In patient safety, this adaptive capacity is conceptualized as resilience-the capacity of a system to adjust its functioning before, during, or after changes to maintain high-quality care (16). Applying this concept to care transitions, resilience-based interventions have sought to strengthen the adaptive capacities of different stakeholders (17, 18).

In addition, differences in knowledge and values of stakeholders, along with differing organizational structures and institutional systems, create boundaries among stakeholders (14). As a result, shared information does not always become shared knowledge; people may interpret the same information differently depending on their expertise and experience (9). Research has shown that when the needs of patients and caregivers are clearly identified and communicated in an understandable and actionable manner, shared understanding and self-care abilities are strengthened (19). These practices are consistent with knowledge brokering, which involves organizing and translating information so that others can use it effectively (20). Hospital nurses, patients, and caregivers have also been recognized as playing knowledge-brokering roles that contribute to safer transitions (8). These findings suggest that interventions focusing on knowledge-brokering functions may help bridge boundaries among stakeholders and organizations, thereby improving the safety of care transitions.

Several instruments have been developed to capture the quality of care transitions from the patient’s perspective. The Care Transitions Measure, developed in the United States, is the most widely used instrument and assesses four domains: information transfer, patient and caregiver preparation, support for self-management, and empowerment to assert preferences, as reported by patients themselves (21). In the United Kingdom, the Partners at Care Transitions Measure was developed to capture both the immediate post-discharge period and patients’ subsequent experience of managing care at home (22). These patient-reported instruments have been valuable for benchmarking the overall experience of care transitions and have since been applied and translated in multiple countries (23). However, because they ask patients and caregivers to rate their impression of the transition, they were not designed to capture the specific sequence of practices through which providers, particularly hospital nurses, identify, organize, and convey information to ensure that it can be understood and actionable for patients, caregivers, and receiving providers. In Japan, a few provider-reported instruments have been developed to evaluate discharge support practices, including anticipating post-discharge problems and promoting self-care. Although these instruments include communication as a component of discharge support, they do not explicitly assess information-sharing practices as a distinct construct (24–26). This gap is also reflected in discharge practice in Japan. Information sharing occurs through discharge summaries, pre-discharge conferences, and discharge education (7). Several studies, however, reported that discharge information often lacked the necessary details or did not adequately reflect the perspectives and needs of patients and caregivers to address expected post-discharge challenges (9, 27). This implies that information transfer alone is insufficient; information needs to be interpreted within a shared understanding of context and situation prior to becoming actionable knowledge (8). Despite this need, we did not identify any instruments specifically measuring information-sharing practices that facilitate shared understanding among stakeholders. Moreover, existing scales measure whether information sharing felt adequate to the recipient, but not how, or how well, providers actually perform the knowledge-brokering practices described above. Therefore, this study aimed to develop a scale to assess nurses’ information-sharing practices during hospital-to-home care transitions for older adults with high healthcare needs, and to evaluate its validity and reliability. This self-reported scale is not intended to measure patients’ or caregivers’ understanding of the information or the overall safety of the care transition. Rather, nursing administrators or hospital educators implement this scale to measure comprehensive and adequate information-sharing practices that are theoretically intended to support shared understanding among stakeholders and adaptation during care transition.

## 2 Methods

### 2.1 Study procedures

The research procedure consisted of two steps: (1) scale item development, and (2) validity and reliability testing of the developed scale.

#### 2.1.1 Scale item development

##### 2.1.1.1 Define the construct of the care transition information sharing practice scale (CT-ISPS)

**Figure 1:**
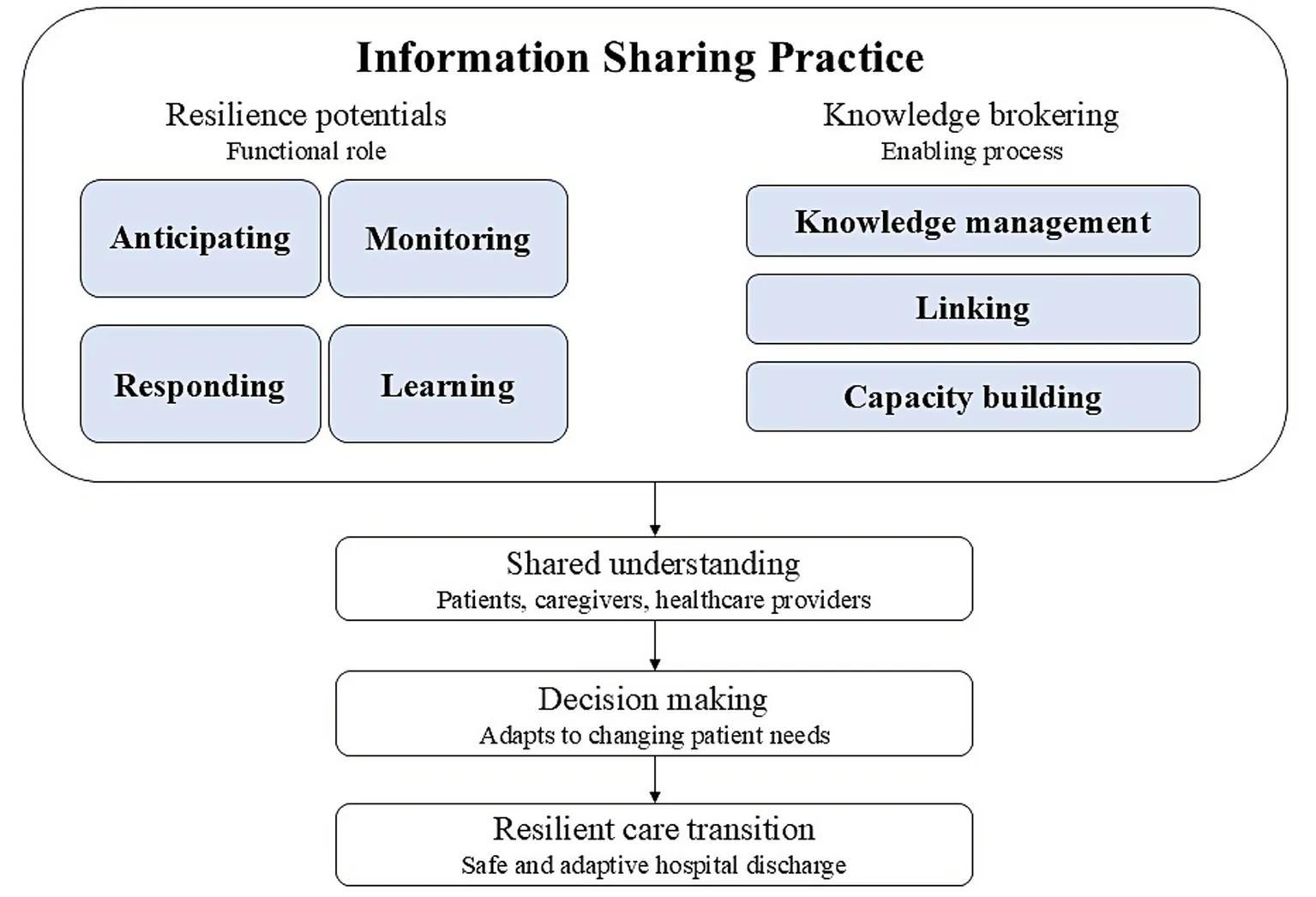
Conceptual framework of the scale.

The purpose of this scale is to measure practices that enable the sharing of essential information to adapt to the changes associated with care transitions. The conceptual framework was developed through a literature review integrating two complementary theoretical perspectives: resilience potential and knowledge brokering (Figure 1). Healthcare resilience informed the functional role of information sharing during care transitions by emphasizing its contribution to four resilience potential—anticipating, monitoring, responding, and learning—that enable care systems involving multiple stakeholders across care settings to adapt to changing patient conditions and care needs (28). The knowledge brokering process informed the processes through which this purpose is achieved, specifically knowledge management, linking, and capacity building to facilitate shared understanding among stakeholders (20). This shared understanding supports decision-making, enabling stakeholders to respond effectively to changes and unexpected situations that arise during the care transition process associated with hospital discharge. These healthcare resilience potentials and knowledge-brokering processes were further used as conceptual domains to guide item generation.

##### 2.1.1.2 Item generation

Scale items representing the construct were generated through (i) a literature review and (ii) consultation with experts.

(i) Literature review

To generate scale items, a literature review was conducted on care transitions, particularly discharge support, drawing on clinical guidelines, existing practice scales, and empirical research. Literature published in English and Japanese was selected using predefined eligibility criteria related to older adults, hospital-to-home transitions, and information sharing. International guidelines and domestic practice measures were searched through Cumulative Index to Nursing and Allied Health Literature (CINAHL) and Ichushi-Web databases (Japan Medical Abstracts Society). Moreover, international studies were identified via PubMed, CINAHL, and MEDLINE. After screening and quality appraisal, 23 sources (3 guidelines, 4 practice measures, and 16 empirical studies) relevant to resilience potentials or knowledge-brokering functions during care transitions were selected for review. The conceptual domains guided the deductive coding of the included literature to identify observable information-sharing practices and ensure comprehensive content coverage. This process yielded 59 candidate practices, which formed the basis for the questionnaire items.

(ii) Expert review

The literature was further reviewed, consolidated, and refined to reflect the Japanese care-transition context. Under the guidance of a nursing faculty member with experience in scale development and through iterative discussions among authors, redundant, low-relevance and overlapping items were removed, and the wording was revised, resulting in 37 preliminary items.

Content validity was then examined through consultations with two master’s-prepared visiting nurses who had ≥10 years of experience in hospital and home care transitions. Visiting nurses were considered appropriate experts because they are highly familiar with post-discharge challenges and are well-positioned to evaluate how information provided by hospitals is utilized in actual care settings. The experts evaluated item relevance, comprehensiveness, and clarity. Each semi-structured interview lasted approximately 40–50 min. As a result, “stakeholders’ role clarification for end-of-life care” was identified as an additional practice and incorporated into an existing item addressing emergency responses, resulting in 13 revisions. One item, “Share information about the patient’s living environment and the care providers to assess the caregiving situation,” was eliminated due to content overlap with the item “Share information on how to monitor the patient’s condition and living situation, including the roles of caregivers in monitoring” (Supplementary Table 1). A final pool of 36 items for the Care Transition Information Sharing Practice Scale (CT-ISPS) was obtained.

##### 2.1.1.3 Item and scale refinement

The 36 items, reflecting resilience and knowledge-brokering functions, were organized into 5 factors and arranged according to the discharge support process used by hospital nurses. Four factors corresponded to the resilience potentials, whereas the fifth, decision-making support, emerged as a critical factor of care transition at discharge: (1) Anticipating life after care transition (16 items; 1–16), (2) care direction and decision-making (4 items; 17–20), (3) learning based on care direction (8 items; 21–27 and 36), (4) continuous monitoring of risks after care transition (5 items; 28–32), and (5) responding to change (3 items; 33–35). The scale used a 5-point Likert format: 1 = Never, 2 = Rarely, 3 = Sometimes, 4 = Usually, and 5 = Always. Study participants were instructed to consider their information-sharing practices at discharge for older adults with high healthcare needs who require frequent medical and nursing care due to complex medical conditions, such as critical or unstable symptoms, the use of medical devices, indwelling catheters, or pressure injuries. Participants rated the frequency of each behavior over the past year. Comprehensibility and feasibility were tested with a nurse experienced in hospital and primary care. Minor wording revisions were made, including clarification of information-sharing modes.

### 2.2 Validation study

#### 2.2.1 Study design and setting

This study employed a nationwide cross-sectional web-based survey to develop and validate a new scale (CT-ISPS). Hospitals with ≥200 beds and general inpatient wards were selected as they frequently provide discharge support to home settings. Facilities of this size were included to ensure sufficient discharge cases and a greater likelihood of employing nurses who work in cross-organizational departments or hold specialized qualifications relevant to the study. To minimize the effects of geographic location and regional characteristics, hospitals listed in the Japan Hospital Association membership directory (29) were stratified into seven geographic regions, and facilities were randomly selected from each stratum. Psychiatric hospitals were excluded due to distinct discharge processes influenced by sociocultural factors.

#### 2.2.2 Study participants

Eligible participants were registered nurses with ≥5 years of experience who were engaged in discharge support through preparation of nursing discharge summaries and/or participation in multidisciplinary pre-discharge conferences, as well as nurses working in cross-organizational departments (e.g., community liaison departments) responsible for discharge planning.

The sample size required for factor analysis depends on various conditions, including the number of scale items and factor loadings. However, when sufficient data are available, the sample size can be calculated at 5–10 participants per item (30). As the CT-ISPS consisted of 36 items, we targeted a minimum calculated sample size of 5 × 36 = 180 (as 5–10) for each of the separate exploratory factor analysis (EFA; n = 180) and confirmatory factor analysis (CFA; n = 180) samples. Based on a previous study and assuming a 10% response rate (31), invitations were sent to 200 hospitals, with each hospital distributing surveys to 20 eligible nurses.

#### 2.2.3 Data collection procedures

Research invitation letters, acceptance forms, participant information sheets, and 20 survey packets were mailed to the nursing administrators of the selected hospitals. When a hospital agreed to participate, the nursing administrator was asked to return an acceptance form by mail to the research team and distribute the participant information sheet to eligible nurses. Participants accessed the web-based questionnaire using the QR code provided on the information sheet. The survey was administered using Microsoft Forms, which provides secure data management. Participants were informed that checking the electronic consent box at the beginning of the questionnaire would constitute informed consent. The questionnaire was designed to take no more than 20 min to complete. The study was anonymous, and no combination of survey items could be used to identify individual participants. Data collection was conducted from November 2024 to February 2025.

#### 2.2.4 Survey measures

The survey included demographic variables (clinical experience, basic nursing education, qualifications, affiliation [department of employment], roles, type of hospital), frequency of participation in discharge conferences, exposure to interprofessional and decision-making support training, use of digital communication tools, and CT-ISPS. For convergent validity, we used a measure of a theoretically related construct, the Interprofessional Collaboration Behavior Scale (ICBS) (32), a validated 17-item, 5-factor scale that measures interprofessional collaborative behaviors for end-stage or medically dependent patients in home care settings. Factor 1, “Decision-making support,” included 4 items; Factor 2, “Sharing predictive judgments,” included 3 items; Factor 3, “Adjustment of care plan,” included 3 items; Factor 4, “Building team relationships,” included 5 items and Factor 5, “24-hour support system,” included 2 items. This scale was selected because it measures behaviors involving anticipation and adaptation to patients’ changing conditions, which are conceptually consistent with the constructs measured by our scale. Items were rated on a 5-point Likert scale assessing self-reported behaviors: 1 = Not at all applicable, 2 = Slightly applicable, 3 = Neither applicable nor inapplicable, 4 = Applicable, and 5 = Highly applicable. Higher scores indicate better interprofessional collaboration behavior.

### 2.3 Data analysis

Data were analyzed using IBM SPSS Statistics Version 22 and AMOS Version 19. Descriptive statistics, including frequencies, proportions, means, and standard deviations (SD), were calculated for all variables. Normality of data distribution was assessed using the Shapiro–Wilk test. In item analysis, the criteria for potential item removal were as follows: floor effects (mean − SD < 1) and ceiling effects (mean + SD > 5), reflecting limited response variability; corrected item-total correlations < 0.20, suggesting poor consistency with the underlying construct; no significant difference (*p* ≥ 0.05) between the lower (≤25th percentile) and upper (≥75th percentile) scores in Good-Poor analysis for item discrimination, and inter-item correlations less than 0.20 suggesting poor homogeneity while proximately 0.80 or higher indicating potential redundancy (33). These criteria were considered alongside the conceptual relevance and content coverage of items when making item-removal decisions.

For factor analysis, we split the participants based on recommendations of a minimum of 5 participants per item; 180 (36 items × 5) for the EFA and the remaining 173 for the CFA. Although a larger CFA sample is generally preferred, allocating more participants to the CFA would have reduced the EFA sample below its recommended minimum. Comparisons of participant characteristics between EFA and CFA were performed using chi-square tests or Fisher’s exact tests. In EFA, the number of factors was determined by Kaiser-Guttman criteria (eigenvalues >1.0) and the scree plot (33). Items with factor loadings <0.40, cross-loadings within 0.20 of the primary loading (34), or poor conceptual interpretability were evaluated for removal (35). Subsequently, CFA was conducted to evaluate construct validity. Model fit indices were evaluated as follows: chi-square/degree of freedom ≤2 as a good fit, ≤3 as acceptable; root mean square error of approximation (RMSEA) ≤ 0.05 as a good fit, ≤0.10 as acceptable. For the goodness of fit index (GFI), comparative fit index (CFI), normed fit index (NFI), relative fit index (RFI), incremental fit index (IFI) and the Tucker-Lewis index (TLI), we considered the value of ≥0.95 as a good fit and ≥0.90 as acceptable (36). Modification indices were examined for potential model misfit, and correlated residuals were specified based on the indices and item content.

Internal consistency was evaluated using Cronbach’s alpha (α), with 0.7–0.9 being desirable. Convergent validity was evaluated by hypothesis testing using the ICBS. We hypothesized a moderate-to-strong positive correlation (Spearman’s *ρ* = 0.60–0.80) between CT-ISPS and Factors 1, 2 and 3 of the ICBS, as they assess related constructs. Spearman’s rank correlation coefficient was employed for the non-normal distribution of data.

## 3 Results

### 3.1 Response rate

A total of 4,000 questionnaires were distributed across 200 hospitals and 64 hospitals indicated their acceptance to participate by returning the acceptance form. A total of 353 valid responses were obtained. Because anonymous distribution was managed locally and the number of questionnaires actually delivered to eligible nurses at each hospital was not recorded, an exact individual-level response rate could not be calculated; 8.8% represents the proportion of valid responses relative to the maximum number of survey packets supplied.

### 3.2 Participant characteristics

As shown in Table 1, participants had 18.9 ± 8.8 years of experience; most worked in general hospitals (71.4%) and general wards (56.4%) and were staff nurses (56.1%). Most participants had interprofessional (70.5%) and decision-making training (62.6%). No significant differences were found between EFA and CFA participants.

**Table 1:** Characteristics of participants (*n* = 353).

Variables	Total (*n* = 353)		EFA (*n* = 180)		CFA (*n* = 173)		χ^2^ (df)	*p*
	*n*	%	*n*	%	*n*	%		
Years of experience as a registered nurse (Mean ± SD = 18.9 ± 8.8 years)								
5–9	54	15.3	29	16.1	25	14.5	0.401 (3)	0.94
10–19	126	35.7	63	35.0	63	36.4		
20–29	121	34.3	60	33.3	61	35.3		
30 and more	45	12.7	24	13.3	21	12.1		
Missing	7	2.0	4	2.2	3	1.7		
Basic nursing education								
3-year vocational program	199	56.4	99	55	100	57.8	5.159 (4)	0.271
4-year University	43	12.2	23	12.8	20	11.6		
3-year college	31	8.8	17	9.44	14	8.1		
5-year integrated program	26	7.4	9	5	17	9.8		
Others	51	14.4	31	17.2	20	11.6		
Missing	3	0.8	1	0.6	2	1.2		
Qualification								
Public Health Nurse (National License)	38	10.8	19	10.6	19	11.0	0.017 (1)	0.897
Certified Nurse Specialist (Master’s-prepared)	23	6.5	11	6.1	12	6.9	0.099 (1)	0.753
Nurse Practitioner (Master’s-prepared)	41	11.6	23	12.8	18	10.4	0.484 (1)	0.487
Certified Nurse	13	3.7	7	3.9	6	3.5	0.440 (1)	0.834
Case Manager (National License)	29	8.2	13	7.2	16	9.2	0.480 (1)	0.488
Affiliation (Department)								
General unit	199	56.4	104	57.8	95	54.9	2.097 (8)	0.978
Working across units in the hospital (i.e., Community Liaison Department)	69	19.5	34	18.9	35	20.2		
Community-based integrated care unit	22	6.2	11	6.1	11	6.4		
Convalescent rehabilitation unit	18	5.1	10	5.6	8	4.6		
Palliative care unit	8	2.3	4	2.2	4	2.3		
Long-term care unit	8	2.3	4	2.2	4	2.3		
Intensive care/High-care unit	5	1.4	3	1.7	2	1.2		
Administrative department	1	0.3	0	0.0	1	0.6		
Others	23	6.5	10	5.6	13	7.5		
Roles								
Staff nurse	198	56.1	101	56.1	97	56.1	2.320 (4)	0.677
Assistant manager/Unit leader	107	30.3	53	29.4	54	31.2		
Unit Manager	30	8.5	16	8.9	14	8.1		
Nursing Administrator/Director	2	0.6	0	0.0	2	1.2		
Others	7	2	4	2.2	3	1.7		
Missing	9	2.5	6	3.3	3	1.7		
Interprofessional teamwork training								
Yes	249	70.5	128	71.1	121	69.9	0.104 (1)	0.747
No	103	29.2	51	28.3	52	30.1		
Missing	1	0.3	1	0.6	0	0.0		
Decision-making support training								
Yes	221	62.6	119	66.1	102	59.0	1.927 (1)	0.165
No	132	37.4	61	33.9	71	41.0		
Participation in the discharge conference								
Always	111	31.4	60	33.3	51	29.5	0.989(3)	0.804
Occasional	166	47	83	46.1	83	48.0		
Rare	50	14.2	26	14.4	24	13.9		
Never	25	7.1	11	6.1	14	8.1		
Missing	1	0.3	0	0.0	1	0.6		
Affiliation: organization								
General Hospital	252	71.4	129	71.7	123	71.1	0.086 (3)	0.993
Regional medical support hospital	65	18.4	33	18.3	32	18.5		
Special functioning hospital	33	9.3	16	8.9	17	9.8		
Others	2	0.6	1	0.6	1	0.6		
Missing	1	0.3	1	0.6	0	0.0		
Digital tool use								
Yes	60	17	35	19.4	25	14.5	1.559 (1)	0.212
No	293	83	145	80.6	148	85.5		

EFA, Exploratory factor analysis; CFA, Confirmatory factor analysis, χ^2^, Chi-square statistic, df: degrees of freedom.

### 3.3 Item analysis

The overall mean score of the preliminary scale was 4.07 ± 0.92. Item means ranged from 3.31 for Item 8, “Share information on alcohol and tobacco use, including non-use”, to 4.63 for Item 5, “Share information on mobility, such as walking and activity level”. Ceiling effects were observed on 18 items; however, these items were retained because of their conceptual relevance to the construct. No floor effects were observed. Corrected item-total correlation coefficients ranged from 0.527 to 0.852, and all items showed significant differences between the high- and low-score groups in the GP analysis. Inter-item correlation coefficients ranged from 0.172 to 0.856. The following 4 items were removed due to correlation indicating potential redundancy with other items: For Item 17 and 19 (ρ = 0.806), Item 17 “Share the preferences of the patient and family regarding information sharing” was removed while Item 19 “Share patients’ and families’ wishes and concerns for future life and treatment” was retained because it addressed the broader wishes of patients and family; For Item 25 and 26 (*ρ* = 0.854), Item 25 “Share simple methods for medication management and medical care that patients and caregivers can perform safely” was removed while Item 26 “Share methods and purpose of medical and self-care with patients and caregivers” was retained because it covers both methods and purpose of care; For Item 30 and Item 31 (*ρ* = 0.789) and Item 32 and 31 (*ρ* = 0.792), both Item 30 “Share timing when problems are likely to occur, such as changes in living environment, medication, or worsening of the disease” and Item 32 “Share appropriate timing and for care providers to review the patient’s health status and living situation” were removed while Item 31“Share information on how to monitor the patient’s condition and living situation, including the roles of caregivers in monitoring” was retained as it provided broader coverage of monitoring for both patient’s condition and living situation, whereas Items 30 and 32 represented more specific aspects of monitoring such as timing. The remaining 32 items were used for the next factor analysis.

### 3.4 Factor analysis

#### 3.4.1 Exploratory factor analysis (EFA)

The 32 items showed factorability (KMO = 0.950; Bartlett’s *p* < 0.01). Maximum likelihood with Promax rotation (Kaiser normalization) yielded a 3-factor solution. Based on predefined criteria, items were iteratively evaluated, and following 9 items were removed: Item 10 “Share information about indication, mechanism of action and use of prescribed medicine”, Item 16 “Share symptoms and conditions requiring palliative care in advanced disease stages”, and Item 21 “Share conditions and reasons underlying the difficulty of remaining at home in past” were removed for low factor loading; and Items 11 “Share information about medication management ability of patient and caregivers,” Item 15 “Share information based on common assessment measures,” Item 18 “Share perception and understanding of patient and family about disease status and treatments,” Item 22 “Share information about the patient’s and caregiver’s physical and psychological readiness for learning and their level of understanding” for cross-loading. Item 23 “Share anticipated challenging situations based on the shared care direction.” was initially retained because of its contribution to the construct but subsequently removed because of its strong correlation with Item 24 (Spearman’s *ρ* = 0.821) and substantial content overlap with other items of the same factor. Lastly, Item 9 “Share information about infection and allergy” for poor interpretability. The final model included 23 items across 3 factors (Table 2).

**Table 2:** Exploratory factor analysis: maximum likelihood with Promax rotation (*n* = 180).

Factors and items (23 items)	Factor loading			Communality
Factor 1 Knowledge sharing to support adaptation (11 items)				
33. Share how to communicate and respond to changes in condition, including end-of-life, with the roles of caregivers and care providers.	0.987	−0.216	0.001	0.736
31. Share information on how to monitor the patient’s condition and living situation, including the roles of caregivers in monitoring.	0.848	−0.009	−0.036	0.667
35. Share how to adjust medication and medical care according to symptoms, based on the ability of the patient and caregivers.	0.761	−0.073	0.178	0.720
27. Ask the recipient of the information to explain in their own words to confirm understanding.	0.759	−0.162	0.062	0.496
28. Share the symptoms and situations that require urgent response, along with the reasons for urgency.	0.709	0.154	−0.024	0.644
34. Share information on medical and long-term care services expected to be needed in the near future.	0.701	−0.098	0.283	0.738
36. Share information using easy-to-understand language and formats.	0.655	0.369	−0.142	0.710
29. Share nonspecific symptoms, such as declines in activities of daily living or appetite, and changes in cognitive function or consciousness, along with the reasons to monitor such symptoms.	0.654	0.284	−0.067	0.676
26. Share methods and purpose of medical and self-care with patients and caregivers.	0.61	0.303	−0.151	0.546
24. Share practical strategies developed together with the patients and caregivers to prevent anticipated problems.	0.548	0.129	0.269	0.740
20. Share care providers’ views, such as an attending doctor’s, on the disease stage and treatment plan.	0.491	0.018	0.237	0.483
Factor 2 Sharing patient’s holistic status to set directions (8 items)				
7. Share information on elimination (bladder and bowel function).	−0.147	0.966	−0.073	0.696
5. Share information on mobility, such as walking and activity level.	−0.304	0.899	0.163	0.673
3. Share information on cognitive function and behavioral and psychological symptoms, including their absence.	0.105	0.819	−0.093	0.696
6. Share information on dietary and fluid intake and swallowing function.	0.067	0.772	0.008	0.679
1. Share physical symptoms and their underlying pathology that are likely to affect daily life.	−0.005	0.701	0.013	0.499
4. Share information on sensory functions such as vision and hearing.	0.233	0.533	0.027	0.533
2. Share psychological symptoms such as anxiety and depression, including their absence.	0.184	0.464	0.113	0.473
19. Share patients’ and families’ wishes and concerns for future life and treatment.	0.254	0.406	0.279	0.690
Factor 3 Sharing external factors for anticipation (4 items)				
13. Share information on financial situation and public services like long-term care services.	−0.031	0.078	0.839	0.753
12. Share information on the living environment and transportation options.	0.069	0.021	0.697	0.580
14. Share information on caregivers’ capacity and their relationship with the patient.	0.127	0.296	0.515	0.707
8. Share information on alcohol and tobacco use (including non-use).	0.275	−0.147	0.467	0.359

		Factor 1	Factor 2	Factor 3	
Eigenvalues		12.519	2.009	1.092	
Factorial correlation matrix	Factor 1		0.672	0.718	
	Factor 2			0.633	

Description of factors: Factor 1 captures integrated resilience-related practices, including monitoring and responding to changes during care transition.

Factor 2 captures the practice of sharing holistic information to understand care needs.

Factor 3 comprises external and contextual factors relevant to adaptation after discharge.

Three identified factors were labeled based on the conceptual framework: (1) “Knowledge sharing to support adaptation,” reflecting practices that augment adaptive capacity for post-transition changes (2) “Sharing patient’s holistic status to set direction” encompassing physical and mental/spiritual factors required to anticipate and set care directions aligned with patient wishes; (3) “Sharing external factors for anticipation” capturing environmental and social context that affect the future status of older adults.

Shapiro–Wilk tests indicated that the factor scores were not normally distributed (all, *p* < 0.05), supporting the use of non-parametric analyses. Descriptive statistics for the 23 final items are presented in Supplementary Table 2. Ceiling effects were observed in 11 items.

#### 3.4.2 Confirmatory factor analysis (CFA)

**Figure 2:**
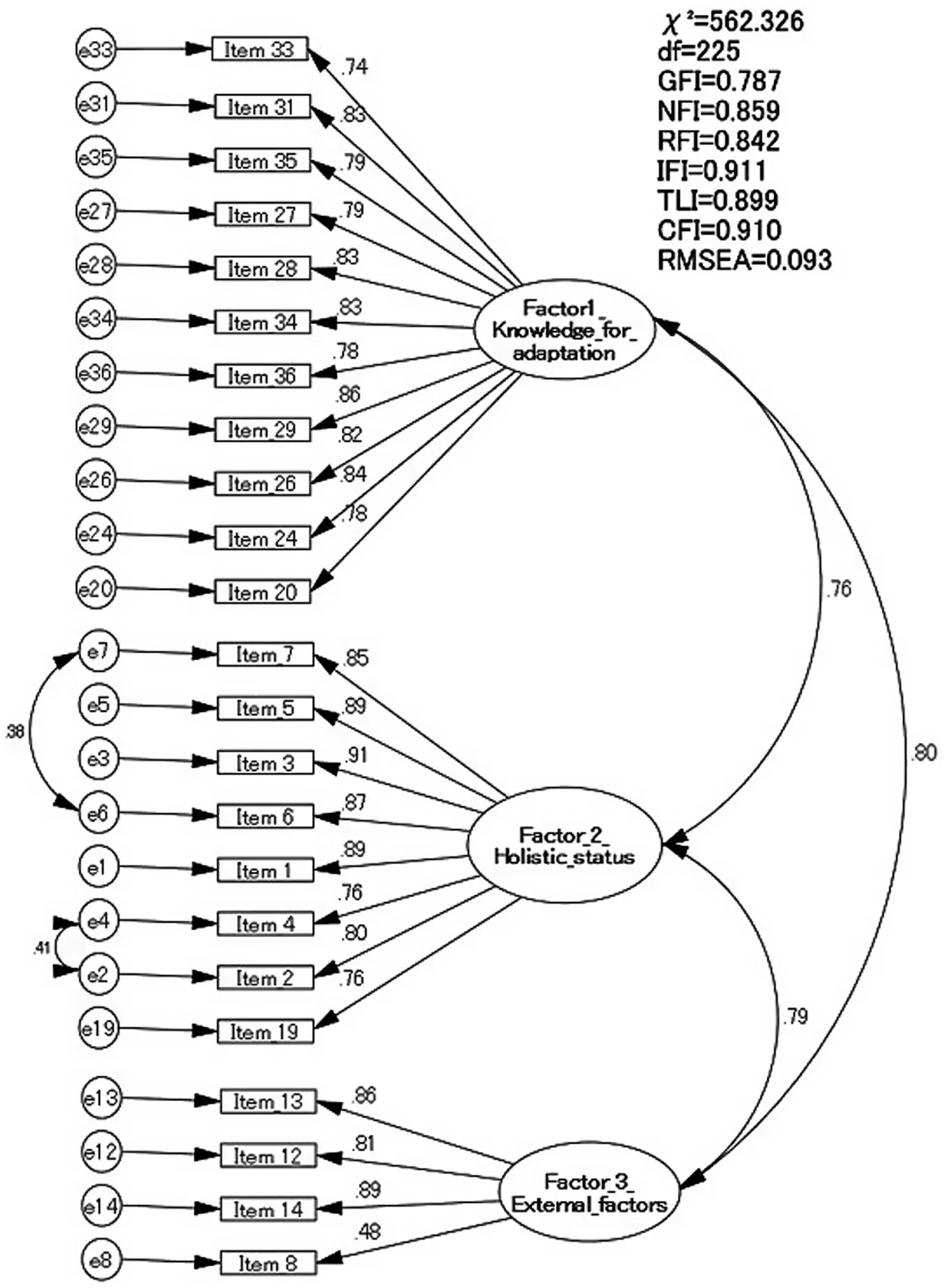
Confirmatory factor analysis path diagram of CT-ISPS (*n* = 173). Standardized factor loadings and factor correlations are rounded to two decimal places; leading zeros are omitted.

A CFA was conducted to examine the fit of the 3-factor structure derived from the EFA. Because multivariate normality was substantially violated (Critical Ratio = 40.40; exceeded the accepted threshold of 5.0), maximum-likelihood CFA was supplemented with Bollen-Stine bootstrapping to obtain a bootstrap-adjusted assessment of overall model fit. Two residual covariances were specified *post hoc* based on modification indices and item-content considerations (Supplementary Table 3). As shown in Figure 2, the model fit indices of the correlated three-factor model were: χ^2^/df = 2.499 (*p* < 0.001), GFI = 0.787, CFI = 0.910, TLI = 0.899, and RMSEA = 0.093 (90% confidence interval: 0.084–0.103). The latent factor correlations were as follows: Factor 1 - Factor 2: 0.76, Factor 1 - Factor 3: 0.80, and Factor 2 - Factor 3: 0.79. To examine unidimensionality, a one-factor model was tested, yielding χ^2^/df = 4.296 (*p* < 0.001), GFI = 0.579, CFI = 0.799, TLI = 0.777, and RMSEA = 0.138. The second-order model, in which the three first-order factors loaded onto a single higher-order factor, yielded identical fit indices to the correlated three-factor model. Additional post-hoc EFA analyses explored item-level refinement, and we evaluated the resulting models using CFA. The modified models 1–3 yielded CFI values of 0.904, 0.915, and 0.917 and RMSEA values of 0.100, 0.096, and 0.097, respectively (Table 3).

**Table 3:** Summary of confirmatory factor analysis models and model fit (*n* = 173).

	Items	Factors	Correlated residual	χ^2^/df	GFI	NFI	RFI	IFI	TLI	CFI	RMSEA
Correlated three-factor	23	3	2	2.499	0.787	0.859	0.842	0.911	0.899	0.910	0.093
One-factor	23	1	2	4.296	0.579	0.755	0.728	0.801	0.777	0.799	0.138
Second-order	23	3 + 1	2	2.499	0.787	0.859	0.842	0.911	0.899	0.910	0.093
Modified 1	22	2	3	2.715	0.776	0.857	0.839	0.905	0.892	0.904	0.100
Modified 2	21	2	3	2.571	0.802	0.870	0.852	0.916	0.904	0.915	0.096
Modified 3	20	2	3	2.622	0.809	0.873	0.854	0.917	0.905	0.917	0.097

Modified model 1 removed Item 8 because of low communality (0.359). Modified model 2 additionally removed Item 14 due to cross-loading (a difference from the primary factor of 0.07). Modified model 3 additionally removed Item 34 because of its moderately strong correlation with Item 33 (*r* = 0.785) and conceptual similarity.

Modification details are provided in the Supplementary Table 3.

### 3.5 Reliability

Cronbach’s alpha coefficients were 0.95 for Factor 1, 0.95 for Factor 2, and 0.83 for Factor 3, indicating high internal consistency. Corrected item-total correlation ranges were: Factor 1 (0.70–0.81), Factor 2 (0.71–0.87), and Factor 3 (0.51–0.75) (Supplementary Table 2). Deletion of any individual item did not substantially alter Cronbach’s alpha. Inter-item correlation ranges were Factor 1 (0.53–0.75), Factor 2 (0.55–0.80), and Factor 3 (0.40–0.74) (Supplementary Table 4).

### 3.6 Convergent validity: correlations of CT-ISPS with the Interprofessional collaboration behavior scale (ICBS)

Spearman’s rank correlation coefficients were calculated for total and factor scores of both scales. As shown in Table 4, the scale total scores of the CT-ISPS and ICBS were strongly correlated (ρ = 0.795). At the factor level, strong correlations (ρ ≥ 0.7) were observed between CT-ISPS Factor 1 and ICBS Factors 2 and 3, and between CT-ISPS Factor 3 and ICBS Factor 3. Correlations with ICBS Factor 4 and Factor 5 ranged from 0.416 to 0.559.

**Table 4:** Correlation between two scale factors (n = 353).

Interprofessional Collaboration Behavior Scale (ICBS) (17 items)	CT-ISPS (23 items)			
	Factor 1 (11 items)	Factor 2 (8 items)	Factor 3 (4 items)	Total (23 items)
Factor 1 (4 items)	0.613**	0.588**	0.436**	0.626**
Factor 2 (3 items)	0.706**	0.579**	0.520**	0.698**
Factor 3 (3 items)	0.708**	0.610**	0.868**	0.791**
Factor 4 (5 items)	0.559**	0.481**	0.456**	0.572**
Factor 5 (2 items)	0.560**	0.416**	0.445**	0.548**
Total (17 items)	0.771**	0.651**	0.672**	0.795**

Spearman’s rank correlation coefficient. **p < 0.01.

## 4 Discussion

To the best of our knowledge, CT-ISPS is the first scale to measure nurses’ practices in care transition for older adults with high medical needs. These practices, according to the proposed conceptual framework, are hypothesized to facilitate the formation of actionable knowledge and adaptation to change and unexpected situations following hospital discharge. Grounded in resilience and knowledge brokering concepts, the 23-item, 3-factor scale demonstrated preliminary evidence of structural and convergent validity and adequate internal consistency in this sample of hospital-based nurses.

### 4.1 Suitability of the data

No missing data were observed in the developed scale items, and background variables had less than 3% missingness, indicating good data completeness. Although ceiling effects were identified in 11 items, particularly in Factor 2, these items were retained because they represented fundamental aspects of information-sharing practice. Consequently, score distributions were skewed toward higher values, potentially limiting the scale’s discriminative ability. Furthermore, the influence of these ceiling effects on the factor structure derived from inter-item correlations cannot be entirely ruled out. Despite the observed ceiling effects, all items demonstrated adequate discriminatory power in the G-P analysis, suggesting that the scale was able to differentiate respondents with relatively higher and lower levels of information-sharing practices. No statistically significant difference was found between EFA and CFA.

### 4.2 Validity of the scale

#### 4.2.1 Content validity

The scale items were derived from broader literature and organized deductively around resilience and knowledge brokering, thereby supporting conceptual relevance and comprehensive coverage (33). From a knowledge-brokering perspective, a few items reflected the linkage function, suggesting that the scale captures more knowledge management or capacity building than linkage. Future research should examine the role of linkage in information sharing during care transition.

Although interviews with older adults and their caregivers were not conducted, the literature included studies informed by patient and caregiver/family interviews, and, together with expert interviews, provided additional support for the relevance of items in care transitions. Nevertheless, the small number of experts and the absence of pilot testing limit the strength of content validity evidence and warrant further evaluation.

#### 4.2.2 Construct validity

The EFA supported a three-factor structure, with moderate-to-high factor loadings (0.406–0.987) and minimal cross-loadings, indicating a coherent and interpretable solution. Most communalities exceeded 0.40, supporting adequate representation of item variance. The reduction from 5 preliminary factors to 3 empirical factors may reflect the integrated nature of resilience potentials (37), where learning the current situation, anticipating changes, followed by monitoring and responding to the patient’s changing needs are closely interrelated processes. However, because the third factor comprises only four items, its stability and reproducibility should be confirmed in larger independent samples.

Factor 1 captured integrated resilience-related practices, such as monitoring and response to changes in condition among older adults and reflected the interdependent nature of the four resilience potentials (37). The conceptual relatedness of these practices likely contributed to the high internal consistency, while item-level analyses indicated that no single item disproportionately influenced the reliability estimates. It also reflected knowledge-brokering functions, including translating information into actionable knowledge by explaining rationale and context, and supporting relational coordination through role clarification. Factor 2 reflected core and holistic information about older adults’ conditions, consistent with standard discharge documentation (38). The high mean scores suggest limited discriminative ability, which may stem from the fundamental nature of the underlying factor. Item 19 “Share patients’ and families’ wishes and concerns for future life and treatment” was retained because, together with the clinical and functional status information captured by the other Factor 2 items, it contributes to a comprehensive understanding of care needs that underpins effective information sharing during care transitions. Its moderate factor loading and strong corrected item-total correlation supported its contribution to the factor. A prior study, however, linked insufficient understanding of such information to care discontinuity, underscoring its importance (27). Factor 3 also comprised standard discharge information items that included external and contextual factors relevant to adaptation after discharge. Although Item 8, “Share information on alcohol and tobacco use (including non-use)” may appear less conceptually aligned with other contextual factors, its inclusion may reflect the common practice of documenting alcohol use and smoking as core social background information in clinical documents in Japan, including discharge summaries. However, its moderate factor loading (0.467) and communality (0.359) suggest that its contribution to the factor warrants further validation to clarify its stability and conceptual role. Deleting this item did not improve model fit, supporting its retention despite its weaker psychometric performance.

Although the three-factor model demonstrated acceptable χ^2^/df and incremental fit indices, the elevated RMSEA and lower absolute fit indices indicated that the model may not fully represent the underlying construct. The relatively small sample size and ceiling effects in nearly half of the retained items may have reduced response variability and potentially contributed to the suboptimal model fit. The relatively strong correlations among factors (*r* = 0.76–0.80) indicated that these factors were closely related, reflecting the interconnected nature of information-sharing practices during care transitions. Nevertheless, substantially poorer fit of the one-factor model was consistent with a multidimensional structure. Post-hoc evaluation of modified two-factor models did not demonstrate a clear improvement in overall model fit compared with the original correlated three-factor model. Although the modified models were more parsimonious and some incremental fit indices showed slight improvement, χ^2^/df and RMSEA did not improve despite adding three residual covariances. Because these modifications were data-driven and evaluated in the same CFA sample, the modified models may reflect sample-specific characteristics. Thus, replication in an independent sample is required to confirm their generalizability. Moreover, item removal may reduce the comprehensiveness of practices relevant to the Japanese context. Therefore, the modified two-factor models were not considered preferable to the original three-factor model. Furthermore, the second-order model included only three first-order factors, its higher-order component was just-identified and had identical fit to the correlated three-factor model. Thus the present analysis cannot provide independent evidence whether a higher-order total construct provides a superior representation of the data. Accordingly, the CT-ISPS is primarily interpreted at the factor (subscale) level in this study. Overall, the three-factor structure is theoretically interpretable and preferable to the one-factor model, but its absolute fit is suboptimal and requires evaluation in an independent sample.

Convergent validity was also supported, as hypothesis testing results were consistent with the predefined hypothesis. The relatively high correlations between the CT-ISPS and ICBS are consistent with their theoretical relationship, as both assess practices that support collaborative care, although the ICBS measures general interprofessional collaboration behaviors whereas the CT-ISPS specifically assesses information-sharing practices during care transitions. The total CT-ISPS score correlated strongly with the total ICBS score (*ρ* = 0.795) and ICBS Factor 3 (“Adjustment of care plan”; *ρ* = 0.791). At the factor level, CT-ISPS Factor 3 (“Sharing external factors for anticipation”) showed a particularly high correlation (*ρ* = 0.868) with ICBS Factor 3 (“Adjustment of care plan”), raising concern about potential construct overlap despite their conceptual differences. Because discriminant validity was not evaluated in the present study, the extent to which CT-ISPS Factor 3 represents a construct distinct from the corresponding ICBS factor remains uncertain. Together, these findings provide preliminary evidence for the construct validity of the CT-ISPS while suboptimal fit of the three-factor model indicates that its structural validity requires further evaluation. Future studies should replicate the factor structure in independent and diverse samples and examine alternative models to further evaluate information-sharing practices during care transition.

### 4.3 Reliability of the scale

Cronbach’s alpha coefficients exceeded 0.8 for all factors, and no substantial changes were observed following item deletion. These findings indicate good internal consistency and homogeneity among the items within each factor. However, Cronbach’s alpha values exceeding 0.90 and high corrected item-total correlations indicate redundancy (33). This tendency was particularly evident in Factors 1 and 2. Although the number of items in a scale influences alpha coefficients, the observed high reliability may also reflect conceptual overlap among certain items, as suggested by strong inter-item correlations. Furthermore, item-level analyses indicated that no single item disproportionately contributed to the reliability estimates. The emphasis on comprehensive construct coverage in this initial validation study may have contributed to some item redundancy; however, excessive item overlaps may reduce the instrument’s sensitivity to detect differences in practice and limit conceptual clarity by emphasizing specific practices within the factor. Future studies should examine whether a more parsimonious version can maintain conceptual comprehensiveness and improve psychometric performance.

### 4.4 Scoring and interpretation

For practical use of CT-ISPS, scores may be calculated for each of the three subscales by summing the item scores ranging from 1 to 5, with higher scores indicating more frequent information-sharing practices. No items are reverse scored. The possible subscore ranges are 11–55 for Factor 1, 8–40 for Factor 2, and 4–20 for Factor 3. A total score is not recommended based on the current validation evidence. All items require a response; therefore, no missing item responses are permitted.

### 4.5 Limitations

This study has certain limitations. First, the response rate was low, which introduces potential response bias. The study included only nurses with at least 5 years of clinical experience, and about 60% reported regularly participating in discharge conferences; thus, respondents were relatively experienced and may have been more engaged in discharge support. Moreover, the 12-month recall period may also have introduced recall bias, as respondents relied on general impressions rather than specific episodes of practice, increasing the potential for socially desirable responses. Together, these factors may have contributed to the relatively high item scores, resulting in ceiling effects. Consequently, the findings may not fully represent nurses with lower levels of engagement in discharge support. In addition, the study sample size target was met for the EFA, but smaller than the estimated minimum required for the CFA. This low sample size may affect the stability of the factor structure and generalizability of the study. Generalizability may also be limited because the available records did not allow us to verify the proportion of invited hospitals that participated, preventing assessment of hospital-level selection bias and representativeness. Future research should aim to reduce respondent burden and consider alternative data collection methods; a large sample size and a shorter recall period are recommended.

Second, the use of self-reported data collected from the same respondents at a single time point may have introduced common method bias, inflated inter-item and inter-factor correlations, and affected the estimation of the factor structure. Multiple methods and independent data sources, such as peer ratings or longitudinal designs, should be incorporated in future studies to reduce the potential influence of common methodological bias.

Third, because questionnaire distribution was managed locally and the number of questionnaires delivered to eligible nurses at each hospital was not recorded, we could not calculate an exact individual-level response rate; therefore, we could not assess potential clustering effects.

Fourth, the instrument was validated only among hospital nurses. Although appropriate for the initial phase of instrument development, care transitions involve multiple stakeholders across settings; therefore, further validation in community settings is needed to establish applicability.

Fifth, although the literature review incorporated studies addressing the perspectives of older adults and caregivers, these stakeholders were not directly involved in item development. In addition, expert review was limited to two master’s-prepared visiting nurses, and quantitative content validity indices were not assessed. Therefore, content validity evidence at the item development stage remains preliminary. Future qualitative and quantitative studies involving local stakeholders are needed to strengthen content validity.

Sixth, although the survey instructions specified older adults with high healthcare needs as the target population, participants were asked to report their general information-sharing practices over the preceding year rather than referring to a specific patient. Therefore, the extent to which responses were consistently anchored to patients meeting the study definition cannot be confirmed.

Seventh, given the high correlation between CT-ISPS Factor 3 and ICBS Factor 3, future studies should further examine the discriminant validity of the CT-ISPS using measures of theoretically distinct constructs and formal statistical methods.

Lastly, measurement stability was not assessed; test–retest reliability should be examined to confirm temporal stability.

## 5 Conclusion

This study developed a 23-item, three-factor scale measuring hospital nurses’ information-sharing practices for older adults with high healthcare needs during care transition from hospital to home. The factors included knowledge sharing to support adaptation, sharing the patient’s holistic status to set directions, and sharing external factors for anticipation. This scale addresses an important gap by providing a standardized measure of information-sharing practices during care transitions, grounded in resilience and knowledge brokering theory. The CT-ISPS showed preliminary evidence of construct validity and internal consistency for assessing information-sharing practices among hospital nurses. Although the proposed three-factor structure was theoretically interpretable and preferable to a one-factor structure, its absolute fit indices were suboptimal, highlighting the need for independent replication. Future research should examine its validity among home-care providers and other stakeholders involved in care transitions.

## Data Availability

The datasets presented in this article are not readily available because. The data analyzed in this study are available upon reasonable request from the corresponding authors. Requests to access the datasets should be directed to Chihiro Kimura, chhrkimura@gmail.com.
