# Supplementary material for: Development of a scale to measure nurses’ information sharing practices during care transitions for older adults with high healthcare needs: a cross-sectional study
Source: Front Public Health. 2026 Sep 10;14:1938148. doi: 10.3389/fpubh.2026.1938148 (PMC13600829; doi:10.3389/fpubh.2026.1938148)
Supplement: Supplementary file 1 [file Table_1.docx]

Supplementary Table 1:
Revision (15 items) and deletion (1 item) of the preliminary 37 items to 36 items

| Item number (16) | Original contents | Change | Contents after revision |
| --- | --- | --- | --- |
| 14 | Share information on the relationship between the patient and the care provider, and the level of support they provide. | Revised | Share information on caregivers’ capacity and their relationship with the patient. |
| 17 | Share the patient's and caregiver's thoughts and intentions regarding information sharing. | Revised | Share the preferences of the patient and family regarding information sharing |
| 20 | Share the views of medical professionals, such as the attending physician, regarding the current situation and future treatment plans. | Revised | Share healthcare professionals’ views, such as the attending doctor, on the disease stage and treatment plan. |
| 21 | Share the reasons why it has become difficult to continue living at home to understand the learning needs of the patients and their caregivers, | Revised | Share conditions and reasons underlying the difficulty of remaining at home in the past. |
| 22 | Share information regarding the readiness and understanding of the patient and caregivers regarding new learning. | Revised | Share information about the patient’s and caregiver’s physical and psychological readiness for learning and their level of understanding |
| 23 | Share what we anticipate will be issues from the perspective of the care plan. | Revised | Share anticipated challenging situations based on the shared care direction. |
| 26 | Share the purpose and methods of medical treatment and management to the patient and care providers. | Revised | Share methods and purpose of medical and self-care with patients and caregivers. |
| 28 | Share the reasons for the urgency and how to respond appropriately for symptoms requiring immediate attention. | Revised | Share the symptoms and situations that require urgent response, along with the reasons for urgency. |
| 30 | Observe closely changes in symptoms and living conditions during periods when problems are likely to occur, such as immediately after a transition or when changing medication. | Revised | Share timing when problems are likely to occur, such as changes in living environment, medication, or worsening of the disease. |
| 31 | Share feasible observation methods and the roles of care providers. | Revised | Share information on how to monitor the patient's condition and living situation, including the roles of caregivers in monitoring |
| 33 | When time is limited, share information on how to respond to sudden changes in a patient's condition and how to contact others as a priority. | Revised | Share how to communicate and respond to changes in conditions, including end-of-life, with the roles of caregivers and care providers |
| 34 | Share information for healthcare insurance services and medical expense subsidies that will be needed in the future. | Revised | Share information on medical and long-term care services expected to be needed in the near future. |
| 37 | Share information regarding living environment, hygiene practices, and the status of caregivers to assess the care situation, | Delete |  |
