# Supplementary material for: Development of a scale to measure nurses’ information sharing practices during care transitions for older adults with high healthcare needs: a cross-sectional study
Source: Front Public Health. 2026 Sep 10;14:1938148. doi: 10.3389/fpubh.2026.1938148 (PMC13600829; doi:10.3389/fpubh.2026.1938148)
Supplement: Supplementary file 2 [file Table_2.docx]

Supplementary Table 2: Descriptive statistics of the final 23 items, including mean, SD, median, ceiling effect and corrected item-total correlation (n=353).

| Item Number | Mean | *SD | **Mean  +SD | ***cITC | Percentage of score for each item | | | | |
| --- | --- | --- | --- | --- | --- | --- | --- | --- | --- |
|  |  |  |  |  | 1 | 2 | 3 | 4 | 5 |
| **Factor 1** | | | | | | | | | |
| 33 | 3.76 | 1.13 | 4.89 | 0.76 | 6.2 | 7.6 | 18.4 | 39.4 | 28.3 |
| 31 | 3.80 | 1.03 | 4.83 | 0.80 | 2.8 | 10.5 | 16.4 | 44.5 | 25.8 |
| 35 | 3.83 | 1.04 | 4.87 | 0.80 | 3.7 | 8.5 | 15.9 | 44.8 | 27.2 |
| 27 | 3.63 | 1.06 | 4.68 | 0.73 | 4.0 | 12.2 | 21.2 | 42.5 | 20.1 |
| 28 | 3.96 | 0.99 | 4.94 | 0.79 | 2.8 | 6.5 | 14.2 | 45.0 | 31.4 |
| 34 | 3.86 | 0.97 | 4.84 | 0.81 | 2.0 | 7.4 | 21.0 | 41.6 | 28.0 |
| 36 | 4.15 | 0.84 | 4.98 | 0.76 | 1.7 | 2.8 | 9.9 | 50.1 | 35.4 |
| 29 | 4.10 | 0.94 | 5.04 | 0.81 | 2.5 | 4.5 | 10.5 | 45.0 | 37.4 |
| 26 | 4.05 | 0.91 | 4.96 | 0.76 | 2.5 | 3.1 | 13.9 | 47.3 | 33.1 |
| 24 | 3.96 | 0.96 | 4.92 | 0.81 | 2.5 | 5.9 | 14.7 | 46.5 | 30.3 |
| 20 | 3.95 | 1.04 | 4.99 | 0.70 | 3.7 | 6.5 | 14.4 | 41.4 | 34.0 |
| **Factor 2** |  |  |  |  |  |  |  |  |  |
| 7 | 4.54 | 0.73 | 5.27 | 0.79 | 1.7 | 1.1 | 2.5 | 33.7 | 60.9 |
| 5 | 4.59 | 0.72 | 5.31 | 0.79 | 1.4 | 0.8 | 2.5 | 27.8 | 67.4 |
| 3 | 4.45 | 0.76 | 5.21 | 0.87 | 1.7 | 4.5 | 11.6 | 40.5 | 41.6 |
| 6 | 4.51 | 0.76 | 5.27 | 0.82 | 1.7 | 1.1 | 2.5 | 33.7 | 60.9 |
| 1 | 4.44 | 0.79 | 5.23 | 0.79 | 1.1 | 2.0 | 5.7 | 33.7 | 57.5 |
| 4 | 4.12 | 0.91 | 5.03 | 0.74 | 1.7 | 4.5 | 12.7 | 42.5 | 38.5 |
| 2 | 4.16 | 0.92 | 5.08 | 0.75 | 1.7 | 4.5 | 11.6 | 40.5 | 41.6 |
| 19 | 4.24 | 0.85 | 5.09 | 0.71 | 1.7 | 2.0 | 10.5 | 41.9 | 43.9 |
| **Factor 3** | | | | | | | | | |
| 13 | 4.02 | 1.02 | 5.04 | 0.75 | 3.4 | 5.9 | 12.5 | 41.6 | 36.5 |
| 12 | 3.71 | 1.09 | 4.79 | 0.75 | 3.7 | 13.9 | 14.4 | 44.2 | 23.8 |
| 14 | 4.18 | 0.93 | 5.11 | 0.70 | 2.3 | 4.0 | 10.5 | 40.2 | 43.1 |
| 8 | 3.20 | 1.26 | 4.46 | 0.51 | 9.6 | 24.6 | 20.1 | 27.5 | 18.1 |

*SD: Standard Deviation. **Ceiling effect= Mean+SD ***cITC: corrected Item-Total Correlation (Factor level)
