# Supplementary material for: Development of a scale to measure nurses’ information sharing practices during care transitions for older adults with high healthcare needs: a cross-sectional study
Source: Front Public Health. 2026 Sep 10;14:1938148. doi: 10.3389/fpubh.2026.1938148 (PMC13600829; doi:10.3389/fpubh.2026.1938148)
Supplement: Supplementary file 3 [file Table_3.docx]

Supplementary Table 3: Modification of CFA Models (n=173)

| **Models** | **〈Factor〉**  **item numbers** | **item removal**  **and rationale** | **Correlated residuals** | **Modification index (MI)** | **Substantive justification for residual correlation** |
| --- | --- | --- | --- | --- | --- |
| **One-factor with correlated residuals** | All 23 items |  | Item6-Item7 | 62.387 | (1) Both items are related to basic physiological functioning and daily care needs, |
|  |  |  | Item2-Item4 | 47.51 | (2) Sensory function (Item 4) is closely related to cognitive and emotional status (Item 2) |
| **Three-factor model**  ***primary model** | 〈Factor 1〉  20,24,26,27,28,29,  31 33,34,35,36 (11 items)  〈Factor 2〉 1,2,3,4,5,6,7,19 (8 items)  〈Factor 3〉  8,12,13,14 (4 items) |  | Item6-Item7 | 22.086 | (1) Same |
|  |  |  | Item2-Item4 | 29.258 | (2) Same |
|  |  |  |  |  |  |
| **Second order** | Same as primary model  + higher factor |  | Item6-Item7 | 22.086 | (1) Same |
|  |  |  | Item2-Item4 | 29.258 | (2) Same |
| **Modified 1** | 〈Factor 1〉  12,13,14,20,24,26,27,  28,29,31 33,34,35,36 (14 items)  〈Factor 2〉 1,2,3,4,5,6,7,19 (8 items) | Item 8 for low communality (0.359) | Item2-Item4 | 28.954 | (2) Same |
|  |  |  | Item12-Item13 | 50.863 | (3) Both living environment and transportation (Item 12) and financial situation and public service (Item 13) are related to accessibility in daily life and healthcare. |
|  |  |  | Item13-Item14 | 47.512 | (4) Financial situation and public service (Item 13) and caregivers’ capacity and their relationship (Item 14) both refer to the availability and feasibility of support. |
| **Modified 2** | 〈Factor 1〉  12,13,20,24,26,27,28,  29,31 33,34,35,36 (13items)  〈Factor 2〉 1,2,3,4,5,6,7,19 (8items) | Item 14 for cross-loading (a difference from the primary factor was 0.07) | Item2-Item4 | 28.883 | (2) Same |
|  |  |  | Item12-Item13 | 55.569 | (3) Same |
|  |  |  | Item6-Item7 | 21.501 | (1) Same |
| **Modified 3** | 〈Factor 1〉  12,13,20,24,26,27,  28,29,31,33,35,36(12items)  〈Factor 2〉  1,2,3,4,5,6,7,19 (8items) | Item 34 because of its high correlation with Item 33 (*r*=785). Response to changes (Item33) encompasses the prospective service needs (Item34) | Item2-Item4 | 28.781 | (2) Same |
|  |  |  | Item12-Item13 | 57.107 | (3) Same |
|  |  |  | Item6-Item7 | 21.357 | (1) Same |
