# Supplementary material for: Development of a scale to measure nurses’ information sharing practices during care transitions for older adults with high healthcare needs: a cross-sectional study
Source: Front Public Health. 2026 Sep 10;14:1938148. doi: 10.3389/fpubh.2026.1938148 (PMC13600829; doi:10.3389/fpubh.2026.1938148)
Supplement: Supplementary file 4 [file Table_4.docx]

Supplementary Table 4: Inter-item correlation by factors (n=180).

| **Factor 1** | |  |  |  |  |  |  |  |  |  |  |
| --- | --- | --- | --- | --- | --- | --- | --- | --- | --- | --- | --- |
|  | Item 33 | Item 31 | Item 35 | Item 27 | Item 28 | Item 34 | Item 36 | Item 29 | Item 26 | Item 24 | Item 20 |
| Item33 |  | 0.715 | 0.724 | 0.577 | 0.649 | 0.785 | 0.602 | 0.613 | 0.545 | 0.654 | 0.567 |
| Item31 |  |  | 0.665 | 0.613 | 0.613 | 0.637 | 0.651 | 0.668 | 0.586 | 0.657 | 0.542 |
| Item35 |  |  |  | 0.54 | 0.662 | 0.779 | 0.671 | 0.656 | 0.594 | 0.714 | 0.570 |
| Item27 |  |  |  |  | 0.511 | 0.617 | 0.510 | 0.532 | 0.470 | 0.616 | 0.481 |
| Item28 |  |  |  |  |  | 0.632 | 0.691 | 0.688 | 0.626 | 0.667 | 0.587 |
| Item34 |  |  |  |  |  |  | 0.640 | 0.612 | 0.557 | 0.690 | 0.572 |
| Item36 |  |  |  |  |  |  |  | 0.729 | 0.632 | 0.680 | 0.498 |
| Item29 |  |  |  |  |  |  |  |  | 0.563 | 0.685 | 0.536 |
| Itme26 |  |  |  |  |  |  |  |  |  | 0.674 | 0.514 |
| Item24 |  |  |  |  |  |  |  |  |  |  | 0.599 |

Note. Pearson’s correlation coefficients

| **Factor 2** | |  |  |  |  |  |  |  |
| --- | --- | --- | --- | --- | --- | --- | --- | --- |
|  | Item 7 | Item 5 | Item 3 | Item 6 | Item 1 | Item 4 | Item 2 | Item 19 |
| Item 7 |  | 0.763 | 0.625 | 0.727 | 0.593 | 0.521 | 0.427 | 0.543 |
| Item 5 |  |  | 0.628 | 0.613 | 0.584 | 0.478 | 0.383 | 0.568 |
| Item 3 |  |  |  | 0.701 | 0.558 | 0.669 | 0.694 | 0.654 |
| Item 6 |  |  |  |  | 0.557 | 0.579 | 0.518 | 0.647 |
| Item 1 |  |  |  |  |  | 0.473 | 0.652 | 0.487 |
| Item 4 |  |  |  |  |  |  | 0.638 | 0.566 |
| Item 2 |  |  |  |  |  |  |  | 0.536 |

Note. Pearson’s correlation coefficients

| Factor3 | |  |  |  |
| --- | --- | --- | --- | --- |
|  | Item13 | Item12 | Item14 | Item 8 |
| Item13 |  | 0.65 | 0.736 | 0.454 |
| Item12 |  |  | 0.610 | 0.537 |
| item14 |  |  |  | 0.435 |

Note. Pearson’s correlation coefficients
